# Towards Comparable Microplastic Data: Challenges and Priorities for Environmental Monitoring

**DOI:** 10.3390/toxics14090783

**Published:** 2026-09-04

**Authors:** Marco Parolini

**Affiliations:** Department of Environmental Science and Policy, University of Milan, via Celoria 26, I-20133 Milan, Italy; marco.parolini@unimi.it

**Keywords:** microplastics, environmental monitoring, data comparison

## Abstract

Microplastics (MPs) represent one of the main threats that ecosystems currently have to face. The effects of MPs on ecosystems are not fully understood also because the lack of standardized and harmonized procedures and protocols that allow us to accurately detect and quantify levels of contamination and exposure. Inconsistencies in MP amounts occur because procedural, analytical, and reporting discrepancies notably undermine the comparability of results. This paper summarizes the sources of variability in MP data, appealing to the necessity of standardization and/or harmonization of procedural and analytical methods to avoid misleading comparisons and interpretations.

## 1. Introduction

In the last decade, the pervasive presence of plastics has emerged as a critical and highly debated environmental and public health issue. The growing production and use of plastic materials has resulted in increased emissions and the accumulation of 52.1 Mt/year of large-sized plastic waste (i.e., macroplastics) in the environment [1]. Several monitoring surveys have reported the occurrence of macroplastics in aquatic and terrestrial ecosystems [2,3,4]. It has been estimated that the amount of ocean plastic waste alone increased from 50 Mt in 2015 to 150 Mt by 2025, causing direct or indirect interactions with approximately 700 marine species [5]. In addition, the weathering processes of macroplastics have been found to generate small-sized plastic items, namely microplastics (MP, 1 to <1000 µm, but multiple and inconsistent definitions exist) and nanoplastics (NP, 1 to <1000 nm) [6]; this has emerged as a hot topic in environmental studies [7].

Various monitoring and biomonitoring surveys have highlighted the ubiquitous diffusion of MPs into the atmosphere [8] and abiotic and biotic matrices from marine [9,10], freshwater [11,12], and terrestrial [13] ecosystems, including remote areas such as the deep sea, the Arctic, Antarctica [14,15], high mountains, and glaciers [16,17]. Considering their widespread distribution, environmental persistence, and small size, MPs can easily interact with and be ingested and accumulated by organisms at all levels of the ecological hierarchy, representing a threat to their health.

Exposure to a wide array of microplastics performed under controlled laboratory and in-field conditions resulted in ingestion and accumulation, and ultimately had differing toxic effects on both aquatic and terrestrial and conventional and non-conventional model organisms [18,19], including growth inhibition, oxidative stress, inflammation, cytotoxicity, immunotoxicity, organ damage, and microbial alterations [20]. However, the large variability in response often correlated with contrasting results obtained from different studies, which precluded a univocal outcome on the toxicity of microplastics. Overall, the most common issue in laboratory and field studies on MPs was the assessment of exposure, i.e., MP quantities in exposure media (e.g., water, soil, food), or the amount of MPs ingested and accumulated by organisms. On one hand, the quali-quantitative analysis of MP levels is crucial in field surveys in order to assess their presence, distribution, and fate in different ecosystems. On the other hand, this analysis is fundamental for planning environmentally relevant laboratory experiments aimed at exploring the potential toxicity caused by exposure to uniform items (i.e., the same size, shape, and polymer) or to complex mixtures of differently sized, shaped, and polymer-composed items on opportune model organisms. To date, different procedures, methods, and analytical techniques have been used to measure the presence of MP in environmental matrices, often returning imprecise or incomparable data. Thus, there is an urgent need for the standardization and harmonization of approaches for MP sampling, isolation, and identification in different environmental matrices [21,22,23].

Standardization refers to the application of specific methods according to strict criteria and limited flexibility, which enables the comparability of results between laboratories. Harmonization, on the other hand, ensures that results from different studies are comparable despite variations in methodologies, as long as those methods are rigorously tested. Optimized and validated methods should be comparable to confirm their consistency across institutions, allowing data generated by different, but similar, methods and techniques to be combined. Unlike standardization, harmonization embraces a range of investigatory techniques and requires a minimum set of reportable metrics (e.g., size, shape, and polymer type) and precise reporting of methods to ensure comparability [21]. At present, the scientific literature exhibits confusion and heterogeneity in approaches to measure MP levels in environmental matrices. This overall lack of uniformity arises from various factors, identifiable at all levels of research from sampling to data reporting. For instance, one notable inconsistency lies in the use of different density separation solutions, which vary greatly in efficiency and cost, leading to divergent recovery rates for MPs of different densities. Additionally, detection limits and resolutions vary largely between analytical techniques, affecting the ability to consistently identify and quantify MPs, mainly small-sized ones. These methodological disparities hinder cross-study comparisons and risk underestimating the presence and impact of certain plastic types or sizes. The combination of all of these factors can preclude the comparison of the results from different studies as well as our understanding of the real risk to aquatic and terrestrial organisms resulting from MP exposure.

## 2. Challenges to the Standardization and/or Harmonization of Microplastic Data

Microplastics represent a highly heterogeneous mixture of different polymers, with diverse densities, sizes, and shapes, necessitating careful selection of sampling and processing approaches for their effective identification. There is currently no universally accepted or standardized methodology for their analysis. Consequently, existing reports on MPs have shown large within- and among-matrices variability in the amounts of identified items. The main determinants of this variability can be ascribed to terminology, sample collection, the processing, isolation, and identification of items, and data reporting. For instance, a recent discussion paper highlighted that data variability and underreporting on MP abundance and characteristics stems from a combination of partial evaluation, economic constraints, the recovery efficiency of extraction, the filter pore size used for separation, and the detection limit of the quantification and characterization method used [24].

The development and implementation of a single protocol which works for all matrices is very challenging and almost impossible because of the great variability in the composition of environmental samples, both inorganic and organic, as well as inconsistent access to resources and equipment. For instance, a matrix effect can occur in terms of interferences from co-extracted materials, such as organic matter, minerals, salts, and biofilms, which can affect the detection, identification, and quantification of MPs. Depending on the analytical method, these components may alter polymer degradation or suppress signals during thermal analysis, generate spectral interference in spectroscopic measurements, or trap MPs in complex aggregates, reducing extraction and recovery efficiency [25]. Such effects can ultimately cause both under- and overestimation of microplastic abundance.

**Figure 1:**
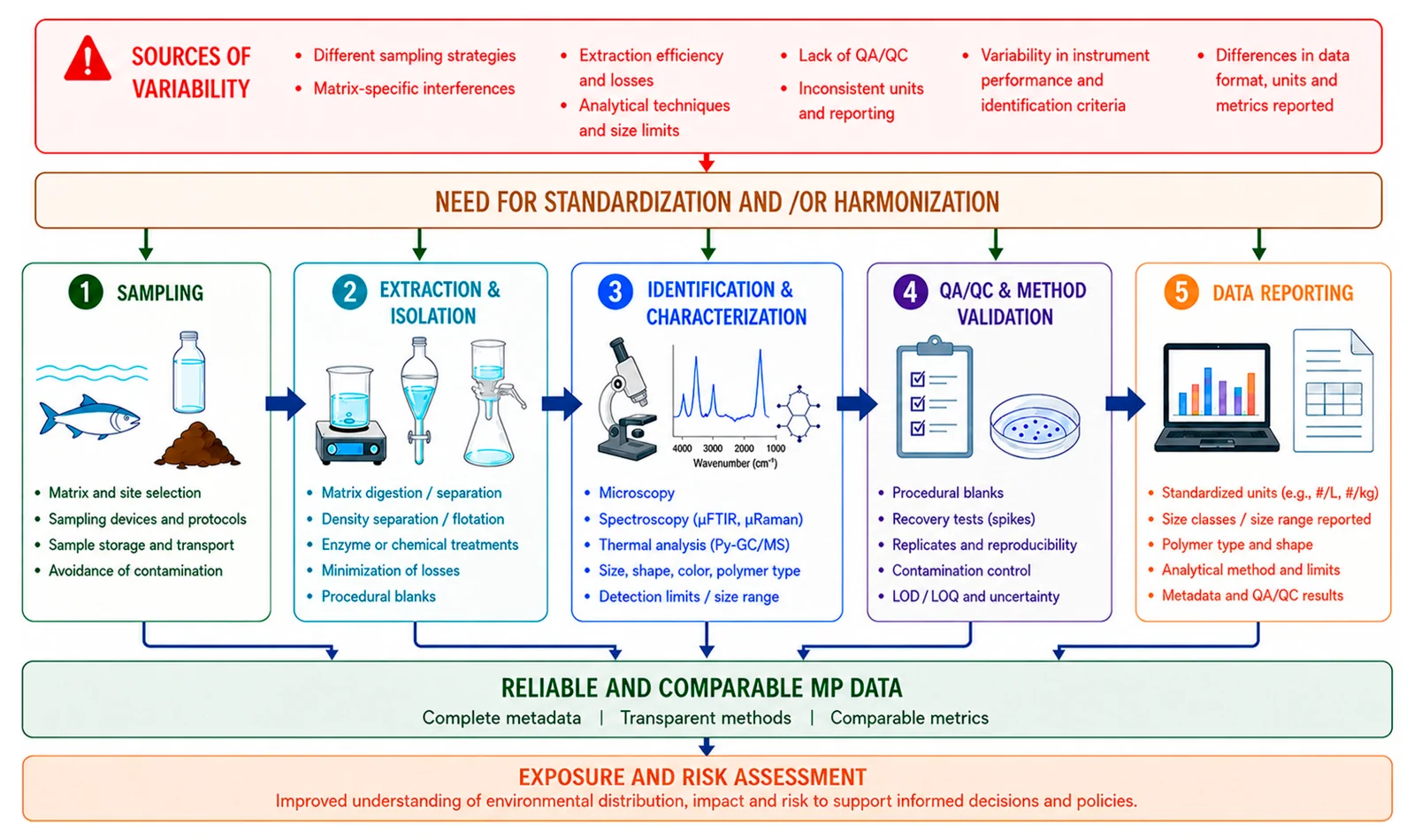
Procedural framework for obtaining comparable microplastic datasets to assess environmental exposure and risk. The symbol ‘#’ in box 5, data reporting, can mean different units, such as items/L, items/kg, mg/L, mg/kg, etc.

Thus, analysis of the current literature and datasets suggests the necessity of guidelines, possibly specific to each environmental matrix, for standardization and/or harmonization of all the steps of microplastic investigation in order to strengthen comparability among studies (Figure 1).

### 2.1. Terminology and Definitions

First, borrowing from the title of the paper by Hartmann and colleagues [26], are we speaking the same language? There is no clear and univocal consensus on the definition and categorization of plastic debris, particularly for MPs. Multiple definitions exist for MPs, which are classified as items in the 1 μm and 5 mm size range [27,28,29], or <5 mm [30]. This lack of a universally adopted size definition leads to confusion and miscommunication, suggesting the importance of adopting a unified terminology for clear definition and categorization when comparing results across studies. Adopting a 1 mm upper size limit may provide greater conceptual and methodological consistency. Indeed, this is a more intuitive boundary following the international system (SI) of units classification to distinguish microscopic fragments from larger visible debris; it aligns with the standard ISO sieve and mesh sizes used routinely in laboratory filtration and physical sortings, and excludes industrial pellets, which are typically 2–5 mm in size and have different origins and environmental fates compared to other MPs [31,32]. This size limit may therefore support clearer classification, greater comparability among studies, and more precise regulatory definitions [32]. This framework goes beyond size classification, but it also includes other features such as physico-chemical properties, shape, colour, and origin as categorization factors.

### 2.2. Sampling and Isolation

Sampling strategies must carefully consider variables such as timing and sample size, ensuring that isolation protocols are as standardized or harmonized as possible within each environmental matrix and, in the case of biota, within each taxonomic group. Sampling designs should be tailored to address the research questions and align with the hypotheses or goals of the survey. These issues should guide decisions regarding sampling locations, equipment, and the number of replicates. Concerning the sample size, although no specific indications on the minimum number of samples is currently available, a recent meta-analysis suggested and recommended a sample size of at least 50 bivalves for MP quantification [33], conforming the value previously suggested by the MSFD Technical Subgroup on Marine Litter [34] and the International Council for the Exploration of the Sea [35]. Temporal and spatial scales must also be considered, as MP distribution can be highly variable across both dimensions. Additionally, selecting an appropriate method to collect the target size range of plastic items and to isolate them from the environmental matrices without altering or damaging is crucial for accurate analysis [36].

Laboratory procedures to isolate MPs from different environmental matrices inevitably depend on the media being studied. Although the National Oceanic and Athmospheric Administration (NOAA) first developed a protocol with specific recommendations for MP analysis in marine waters and sediments [37], several modifications to this protocol have been implemented for the extraction and isolation of plastic items from different environmental matrices [38,39,40,41]. For instance, different salt solutions and purification approaches were proposed and used, with different recoveries of polymers and methods of ‘cleaning’ isolated items [38,39,40]. Moreover, complex matrices such as sediments, soils, or wastewater biosolids can trap tiny plastic items within aggregates, reducing extraction efficiencies during density separation and filtration steps [42]. This wide range of options suggests that there is no ‘right’ method and that method standardization is not currently feasible or advisable [23], but instead that each method must rely on accuracy, precision, and feasibility. In addition, some procedures and analytical techniques intrinsically have a ‘particle size limitation’, precluding the opportunity to analyse items in the sub-micrometer range [43], excluding from consideration small-sized MPs as well as NPs.

### 2.3. Quality Assurance and Quality Control

Another methodological issue concerns quality assurance (QA) and quality control (QC) procedures, which are fundamental in MP investigation [44]. Many studies did not report any method validation or failed to incorporate field and laboratory blanks. These factors can result in potential underestimations of MP levels because of insufficient recovery rates of the employed methods or external contamination during sampling and laboratory procedures, respectively [45,46,47]. For instance, a recent review reported that methods for designing, assessing, and correcting blanks, as well as reporting results, are inconsistent [48]. Thus, blank data results are potentially biased and can introduce unwanted variation in measurements of MPs in environmental samples [49].

However, inter-laboratory studies have shown that despite the potential contamination of samples, underestimations of MP abundance in environmental samples still occur [50]. In addition, in some cases, overestimation of MP levels might result from improper identification of the polymer composition of isolated items, which must be considered as putative MPs until chemical identification. For these reasons, some studies have proposed recalculation [51] and correction [52] factors to assess the data quality of MP abundance. In this context, it is important to distinguish between data accuracy and comparability. Accuracy concerns how reliably measured MP abundance reflects the level of contamination (or exposure), whereas comparability concerns whether results obtained across different studies or analytical approaches can be meaningfully compared. Although closely related, they do not necessarily coincide. Studies applying similar procedures may generate comparable data, while systematically underestimating MP abundance because of low recovery efficiencies or analytical limitations. Conversely, different analytical approaches may provide accurate measurements within their respective operational ranges, yet produce results that are difficult to compare because of differences in particle-size ranges, extraction and isolation procedures, identification criteria, or reporting metrics. For these reasons, QA/QC procedures are primarily required to ensure the accuracy and reliability of MP measurements, whereas standardization and harmonization provide the methodological framework to improve comparability across studies. However, when resources are limited, priority should first be given to achieving a minimum level of accuracy through essential QA/QC measures, including procedural blanks, recovery tests, contamination controls, and confirmation of polymer identity. Comparable but systematically biased measurements may otherwise support incorrect conclusions. Once acceptable accuracy has been demonstrated, resources should be directed toward harmonized sampling, analytical, and reporting procedures to enhance comparability. Of course, priority also depends on the intended application: accuracy is particularly critical for exposure assessment, risk evaluation, and regulatory decisions, whereas comparability is especially important for long-term or large-scale monitoring, as well as trend assessment. Both accuracy and comparability should therefore be addressed in order to generate robust, comparable, and usable MP datasets.

For these reasons, a number of inter-laboratory comparison (ILC) projects (e.g., BASEMAN, Special exercises at WEPAL-QUASIMEME) have been performed or are currently ongoing. These initiatives aim at developing standardized, reliable methods to detect, identify, and quantify MPs in water and other environmental matrices. One example is the inter-laboratory comparison (ILC) involving ISO-approved techniques organized under the pre-standardization platform of VAMAS (Versailles Project on Advanced Materials and Standards) [53]. This project gathered 84 analytical laboratories across the globe to test and compare two thermo-analytical and three spectroscopical methods for MP quali-quantification in a water-soluble matrix using polyethylene terephthalate (PET) and polyethylene (PE) in powder form (10–200 μm size range) as reference materials (RMs) [53]. Thus, ILCs are crucial because they serve as foundational tools for establishing workflow, method validation, QA/QC, identifying sources of inter-laboratory variability, and providing the evidence base required for international standardization and regulatory implementation [54].

### 2.4. Identification Techniques

The identification of MPs in environmental matrices remains highly challenging [49], and the lack of harmonized methodological procedures and common standard parameters often results in underestimation of their abundance. This inconsistency also affects laboratory studies, hindering the quantification of environmental levels, data comparison across matrices or ecosystems, and accurate assessment of organism exposure. Typically, identification of MPs relies on microscopy, vibrational spectroscopy, and thermo-analytical and chromatographic methods coupled with mass-spectrometry [55].

Optical microscopy is a valuable tool for providing insights into the shape and colour of plastic items, but its resolution is limited and ineffective for small-sized MPs. Moreover, the application is labor-intensive, prone to human error, and inadequate for distinguishing plastics from similar-looking inorganic and organic materials [55].

Fluorescent staining (e.g., with Nile Red dye) has been employed to detect MPs through fluorescence microscopy, but encounters difficulties in its adsorption into fibres, the weak fluorescence of certain polymers, and fluorescent contaminants [48]. Scanning and transmission electron microscopy provide details on the size, shape, and surface properties of MPs, but vibrational spectroscopy is required to confirm their polymer composition [55]. Conventional Fourier-Transform Infrared Spectroscopy (FTIR)-based techniques are well-established and non-destructive, but largely ineffective for identification of items smaller than 20 µm [55]. Additionally, the analysis of FTIR spectra is time-consuming, operator-dependent, and bias-prone, as confirmed by a recent study showing that a machine-learning approach outperforms humans in MP characterization [56]. To address this size limitation, microscopy combined with Raman spectroscopy offers an alternative for identifying particles as small as 1 µm, although auto-fluorescence and impurities frequently interfere with detection [55]. Thermal analysis methods, such as pyrolysis (Py) and thermogravimetric analysis (TGA), coupled with mass spectrometry, have emerged as valuable approaches due to their minimal sample preparation, independence from particle size and shape, and ability to characterize complex items and determine organic contaminants. Pyrolysis coupled with gas chromatography and mass spectrometry (Py-GC-MS) is the most widely used approach, although TGA-based techniques represent a promising advancement, but are still underused in the analysis of MPs in environmental samples [57].

Regardless of the analytical technique used for polymer identification, it is important to consider potential matrix effect interference that can affect the quali-quantification of MPs. Indeed, during destructive or thermal techniques, mineral phases (e.g., specific clays or iron oxides) can catalyze early polymer degradation, retain hydrocarbon breakdown products, or cause signal loss, leading to under- or overestimation of specific polymers such as PE, PET, or PVC [25]. In spectroscopic methods (like FTIR or Raman microscopy), dense organic or biological coatings (biofilms) and particulate debris create high background fluorescence, light scattering, or physical obscuration, hiding small MPs or causing false positive/negative polymer identifications [42]. Thus, using optimized digestion (enzymatic, oxidative, or alkaline) and density separation protocols helps to strip away interfering organic and inorganic loads before instrumental analysis [25,42].

Lastly, particle counts alone may inadequately represent MP contamination because they are not conserved during fragmentation and can disproportionately emphasize smaller particles [58]. Mass estimates can therefore complement number-based data and facilitate comparisons of environmental concentrations [59]. In particle-based spectroscopic methods, two-dimensional measurements can be converted into three-dimensional volumes using geometric models; particle mass is then estimated by multiplying volume by the density of the identified polymer [58]. Early approaches used simple shape-specific approximations, such as spheres, cylinders, ellipsoids, or parallelepipeds, whereas recent models accounted for particle morphology and improved the conversion of two-dimensional projections into volume without requiring particle-specific calibration [60]. These approaches enable researchers to obtain particle number, polymer identity, size distribution, and estimated mass from the same analysis. However, mass estimates remain sensitive to assumptions concerning particle shape, thickness, porosity, weathering, and polymer density. Their associated uncertainty should therefore be quantified and reported; indeed, one recent work estimated an additional systematic error of approximately 50% for number-to-mass conversion in atmospheric samples [61].

Mass concentration and mass content may thus provide useful complementary metrics for expressing MP contamination and comparing environmental levels across different samples and studies. Unlike particle count, mass is conserved during physical fragmentation and is therefore less affected by changes in particle number resulting from physical breakdown [59]. Nevertheless, mass and particle counts cannot be considered as interchangeable. Indeed, data counts are strongly influenced by smaller particles, whereas mass-based measurements tend to be dominated by larger particles [59]. Whenever possible, both metrics should therefore be reported together with the analyzed size range. Studies should also clearly distinguish directly measured mass from mass estimated using particle dimensions, geometric assumptions, and polymer density.

### 2.5. Data Reporting

The application of all the techniques mentioned above typically returned data in a range of units. Different units were used to express the levels of MP in abiotic and biotic matrices collected in the real-world environment (e.g., number of items or mass per m^2^ or km^2^ and/or m^3^). Similarly, different units were used to express the exposure levels administered to aquatic and terrestrial organisms in toxicity assays performed under controlled laboratory conditions (e.g., number of items/individual; number of items/volume or weight; concentration–mg/L or µg/mL or amount–mg/Kg or % on weight of solid medium), complicating the comparison of results obtained from different studies. Such heterogeneity in reporting units substantially limits direct comparisons among studies and complicates the integration of data across environmental matrices, geographical areas, and experimental settings.

However, harmonized reporting should extend beyond the use of consistent units. Differences in particle-size ranges, detection limits, and recovery efficiencies can limit data comparability even when the same units are used. Thus, studies should also report the method detection limit and/or the minimum particle size that can be detected and identified. Without this information, zero or low MP concentrations cannot be interpreted reliably, as they may reflect analytical limitations rather than the actual absence or scarcity of items.

Reporting should therefore include the key methodological and analytical information needed to assess data quality, representativeness, and comparability. A minimum reporting set for environmental MP monitoring should include, whenever applicable, the following:

(1) Investigated environmental matrix and the amount of material sampled, expressed as volume, mass, or surface area;
(2) Sampling strategy and number of replicates;
(3) Lower and upper particle-size limits considered throughout sampling and analysis;
(4) Size classes used for data reporting;
(5) MP characteristics, including shape and, when relevant, colour;
(6) Polymer composition, analytical technique used for polymer identification, and information on the polymer identification confidence level (e.g., match quality, number of matching peaks);
(7) Proportion of visually identified particles subjected to chemical confirmation when only a subset is analysed;
(8) Extraction and recovery efficiency;
(9) Field and laboratory blank results and, eventually, the procedure used for blank correction;
(10) Primary unit used to express MP abundance or concentration.

Reporting these parameters would also facilitate the conversion of results into alternative units when sufficient information is available, thereby improving the integration of datasets generated using different approaches. Specifically, particle-size range and polymer identification should be clearly reported, as both can strongly influence MP abundance estimates and data comparability. Visual identification should also be clearly distinguished from analytical confirmation. Minimum reporting requirements may provide a more pragmatic way to harmonization than adopting a single analytical procedure. Transparent reporting of key methodological and analytical information should improve MP data comparability and interoperability, supporting meta-analyses and large-scale assessments of contamination, exposure, and ecological risk.

### 2.6. Environmental Risk Assessment of MP

The ecological risk assessment (ERA) of MPs remains constrained by limited knowledge of their diverse properties (i.e., types, shapes, size, polymer), environmental fate, toxicity, and ecological effects [62]. Available toxicity data are insufficient and largely derived from laboratory studies using a very limited array of commercially produced particles, predominantly PS beads, that poorly represent environmental MPs [29], or otherwise derived from particles produced through different procedures to what is considered standard [63,64]. Consequently, the traditional risk quotient (RQ) method based on chronic toxicity data from species sensitivity distribution (SSD) curves cannot be applied because of the difficulty in introducing accurate hazardous concentration values and appropriate assessment factor values, as well as in calculating the predicted no-effect concentration (PNEC) of MPs in real ecosystems. Some previous investigations have suggested that the application of the RQ approach may underestimate the real risk because of the heterogeneity of MPs in the environment and the limitations of the types of MPs in laboratory testing [65,66,67]. For these reasons, different approaches for the ERA of MPs have been recently implemented. For instance, the Pollution Load Index (PLI), the Polymer Hazard Index (PHI), and the Potential Ecological Risk Index (PERI) are standard metrics which are usually used in combination to evaluate the environmental burden, chemical toxicity, and ecological danger of MPs in aquatic ecosystems [68]. PLI measures the overall contamination level and concentration intensity of MPs relative to a background or baseline (least-contaminated) site, PHI measures specific chemical and ecotoxicological hazard posed by the types of plastic polymers present in the environment, and PERI measures comprehensive, cumulative ecological risk by combining localized abundance with polymer-specific toxicity coefficients [68 and references therein]. The combined use of these indexes should provide a comprehensive, multi-dimensional risk profile that a single index cannot achieve on its own. Indeed, a single index can point to different conclusions (e.g., a site might have low particle abundance but a very dangerous polymer type). Combining different indexes can solve these inconsistencies by clearly distinguishing whether a site’s risk is driven by high MP loads (PLI-driven), hazardous polymer chemistry (PHI-driven), or combined ecological toxicity (PERI-driven) [69]. This multi-index framework allows performing clear risk-tier stratifications to identify exact pollution sources, prioritize mitigation efforts, and communicate ecological risks across different locations [70]. Thus, standardization or harmonization of MP protocols should create a foundation upon which a solid dataset can be built for a reliable ERA.

## 3. Conclusions and Recommendations

Both the standardization and harmonization of procedures and analytical techniques are crucial steps towards obtaining more reliable and comparable data in MP research [24]. While studies using various protocols have advanced this field, the lack of methodological consistency hinders a comprehensive understanding of MP pollution. This variability makes it difficult to accurately assess the extent of contamination, compare findings across studies, and ultimately develop effective mitigation strategies. Pressing issues include inconsistent data reporting, erroneous, incomplete and/or non-comparable datasets, and underestimating or overestimating the toxicity and the fate and distribution of MP. These issues can severely limit the accuracy of toxicological, ecotoxicological, and ecological risk assessments, and hinder informed policy decisions. A number of recent research studies have recommended standardized protocols for MP analysis in environmental matrices [71], including bivalves [33] and riverine matrices [72]. Another possible solution for compiling standardized data was proposed by building a standardization model based on in situ experiments and statistical analysis that can convert existing monitoring data from various sampling approaches into a homogeneous global dataset [36].

Recognizing this critical need, the European Union has taken a significant step towards standardization by tasking a group of experts with developing a standardized method for MP analysis in drinking water [73]. This initiative, a supplement to EU Directive 2020/2184, aims to improve knowledge about MPs in the water supply chain and ensure high-quality drinking water. The EU’s action highlights the importance of robust scientific data in supporting policies that address pressing environmental concerns like MP pollution.

For all the reasons mentioned, efforts to improve the reliability and comparability of MP data should focus on a number of key priorities. First, a common terminology and categorization framework should be adopted to ensure that studies refer to comparable MP particle-size ranges and characteristics. Second, validated and matrix-specific procedures should be developed and, where possible, subjected to inter-laboratory evaluation and calibration rather than pursuing a single analytical protocol. Third, rigorous QA/QC procedures, including procedural blanks, recovery tests, and method validation, should become a pivotal part of MP investigations. Fourth, lower and upper particle-size limits and polymer identification criteria with detection limits should always be explicitly reported, as these factors strongly influence measured MP abundance. Finally, a minimum set of reporting requirements should be established, including information on the investigated matrix, sampling effort, particle-size distribution, polymer composition, recovery efficiency, eventual blank correction, and reporting units. Moreover, future reporting frameworks should also consider the combined use of number- and mass-based metrics, whenever analytically feasible, to support a more comprehensive interpretation of MP contamination and improve comparisons of environmental levels across studies. Particular attention should be paid to the reporting of the approach used for mass determination, thereby ensuring that the resulting data can be appropriately interpreted and included in monitoring and ERA frameworks.

Overall, these priorities support a framework combining the standardization and/or harmonization of procedures in order to obtain robust and comparable datasets for MP risk assessment.

## Data Availability

No new data were created or analyzed in this study. Data sharing is not applicable to this article.

## Abbreviations

The following abbreviations are used in this manuscript:

FTIR	Fourier-Transform Infrared Spectroscopy
MP	Microplastics
NP	Nanoplastics
Py-GC-MS	Pyrolysis coupled with gas chromatography and mass spectrometry
QA	Quality assurance
QC	Quality control
TGA	Thermogravimetric analysis
